# Depletion of the upper mantle by convergent tectonics in the Early Earth

**DOI:** 10.1038/s41598-021-00837-y

**Published:** 2021-11-02

**Authors:** A. L. Perchuk, T. V. Gerya, V. S. Zakharov, W. L. Griffin

**Affiliations:** 1 Faculty of Geology, Lomonosov Moscow State University, Moscow, 119234 Russia; 2 Korzhinskii Institute of Experimental Mineralogy, Russian Academy of Sciences, Chernogolovka, 142432 Russia; 3 Department of Earth Sciences, Swiss Federal Institute of Technology Zurich, Sonneggstrasse 5, 8092 Zurich, Switzerland; 4 Australian Research Council Centre of Excellence for Core to Crust Fluid Systems/GEMOC, Macquarie University, Sydney, NSW Australia

**Keywords:** Geodynamics, Petrology

## Abstract

Partial melting of mantle peridotites at spreading ridges is a continuous global process that forms the oceanic crust and refractory, positively buoyant residues (melt-depleted mantle peridotites). In the modern Earth, these rocks enter subduction zones as part of the oceanic lithosphere. However, in the early Earth, the melt-depleted peridotites were 2–3 times more voluminous and their role in controlling subduction regimes and the composition of the upper mantle remains poorly constrained. Here, we investigate styles of lithospheric tectonics, and related dynamics of the depleted mantle, using 2-D geodynamic models of converging oceanic plates over the range of mantle potential temperatures (T_p_ = 1300–1550 °C, ∆T = T − T_modern_ = 0–250 °C) from the Archean to the present. Numerical modeling using prescribed plate convergence rates reveals that oceanic subduction can operate over this whole range of temperatures but changes from a two-sided regime at ∆T = 250 °C to one-sided at lower mantle temperatures. Two-sided subduction creates V-shaped accretionary terrains up to 180 km thick, composed mainly of highly hydrated metabasic rocks of the subducted oceanic crust, decoupled from the mantle. Partial melting of the metabasic rocks and related formation of sodic granitoids (Tonalite–Trondhjemite–Granodiorite suites, TTGs) does not occur until subduction ceases. In contrast, one sided-subduction leads to volcanic arcs with or without back-arc basins. Both subduction regimes produce over-thickened depleted upper mantle that cannot subduct and thus delaminates from the slab and accumulates under the oceanic lithosphere. The higher the mantle temperature, the larger the volume of depleted peridotites stored in the upper mantle. Extrapolation of the modeling results reveals that oceanic plate convergence at ∆T = 200–250 °C might create depleted peridotites (melt extraction of > 20%) constituting more than half of the upper mantle over relatively short geological times (~ 100–200 million years). This contrasts with the modeling results at modern mantle temperatures, where the amount of depleted peridotites in the upper mantle does not increase significantly with time. We therefore suggest that the bulk chemical composition of upper mantle in the Archean was much more depleted than the present mantle, which is consistent with the composition of the most ancient lithospheric mantle preserved in cratonic keels.

## Introduction

**Figure 1:**
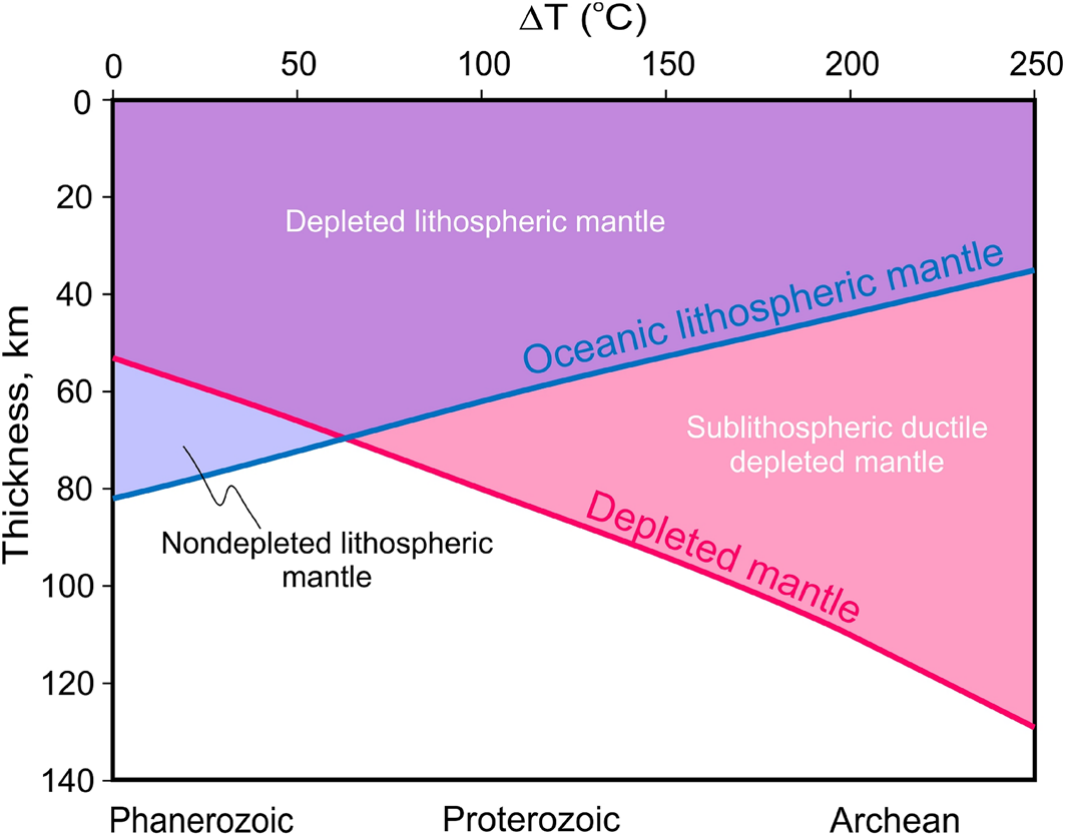
Divergent thicknesses of the lithosphere and depleted mantle (restites) at different potential mantle temperatures (T_p_). Note the considerable protrusion of the depleted mantle through the lithosphere in Archean time (at ΔT > 1500 °C) and hidden within the lithosphere in the modern Earth. Thicknesses of the lithosphere and depleted mantle were taken directly from the output file related to the very initial stage of the modeling and correspond to isotherm 1300 °C and 20% of mantle melt-depletion, respectively.

Global heat balance between internal heating and surface heat loss, and the compositional variations of mantle melts through time provide evidence that the early Earth was hotter than at present^1,2^. Higher mantle temperatures produce thinner lithosphere, increase the degree of melting and decrease the viscosity of mantle peridotites^3–5^ thus producing thicker oceanic crust and complementary melt-depleted peridotite residues (restites) (Fig. 1). All these features are crucial for early-Earth lithospheric and mantle dynamics and related processes, which remain poorly understood.

Petrological studies have shown that Precambrian metamorphic terrains record two contrasting types of metamorphism (intermediate vs high thermal gradients), which can be traced back to ca. 2.8 Ga^6^. This “paired” metamorphism, comparable to Phanerozoic subduction record, has been interpreted to reflect Precambrian one-sided subduction at convergent plate boundaries^6^. However, subduction-diagnostic rock assemblages such as blueschists, low-temperature eclogites and ultra-high pressure metamorphic rocks are restricted mainly to Phanerozoic terrains^7,8^. This implies that subduction and global tectonic style(s) have changed through time^9–13^, although there is also the issue of preservation in such ancient rocks.

The styles of early-Earth tectonics can be predicted from geodynamic modeling, and tested against the geological record^14–18^. Modeling of subduction in ocean–continent and ocean–ocean settings indicates that plate convergence above fertile upper mantle would not have produced self-sustaining subduction before the Paleoproterozoic at mantle temperatures > 175 °C above the present (∆T = 175 °C) because the plates are weakened by melt percolation^19,20^. Numerical models also suggest that two-sided subduction at Archean mantle temperatures can produce pressure–temperature (P–T) regimes that resemble present-day plate tectonic environments^11^. The modeled evolution of such relatively warm and weak lithospheric plates, producing symmetrical two-sided subduction, bears many similarities to other 2-D and 3-D numerical models of Archean tectono-magmatic processes^16,18,21–23^ They define a pre-plate tectonic regime of internally deformable lithosphere, termed squishy-lid^16^, mobile-lid^24^ or plume-lid tectonics^22,25^.

Recent numerical modeling also suggests that dynamics of refractory and positively-buoyant depleted mantle play a crucial role in formation of the lithospheric mantle (mantle keels) beneath cratons in Precambrian *oceanic-continental* subduction/collision zone settings^17^. In this paper, we systematically investigate the formation and dynamics of the depleted mantle in another (*intra-oceanic*) key convergent plate environments using petrological-thermomechanical numerical modeling approach^17^, which accounts for both tectonic and magmatic processes. We have designed a new regional two-dimensional (2-D) high-resolution thermal–mechanical model of oceanic convergence (“Method”, Supplementary Fig. S1, Supplementary Data Table S1). At present-day mantle temperature conditions, we chose a set of reference model parameters that allows satisfactory reproduction of modern styles of subduction. The model is then extrapolated to Precambrian conditions by changing mantle potential temperature and crustal thickness (“Method”). Our numerical results illustrate the crucial role of mantle melt-depletion in Archean lithospheric and mantle dynamics, affecting plate convergence styles over a wide range of mantle temperatures (Supplementary Table S1) and secular changes in both lithospheric and asthenospheric upper-mantle composition during Earth’s evolution.

## Results

### One-sided subduction at ∆T ≤ 200 °C

**Figure 2:**
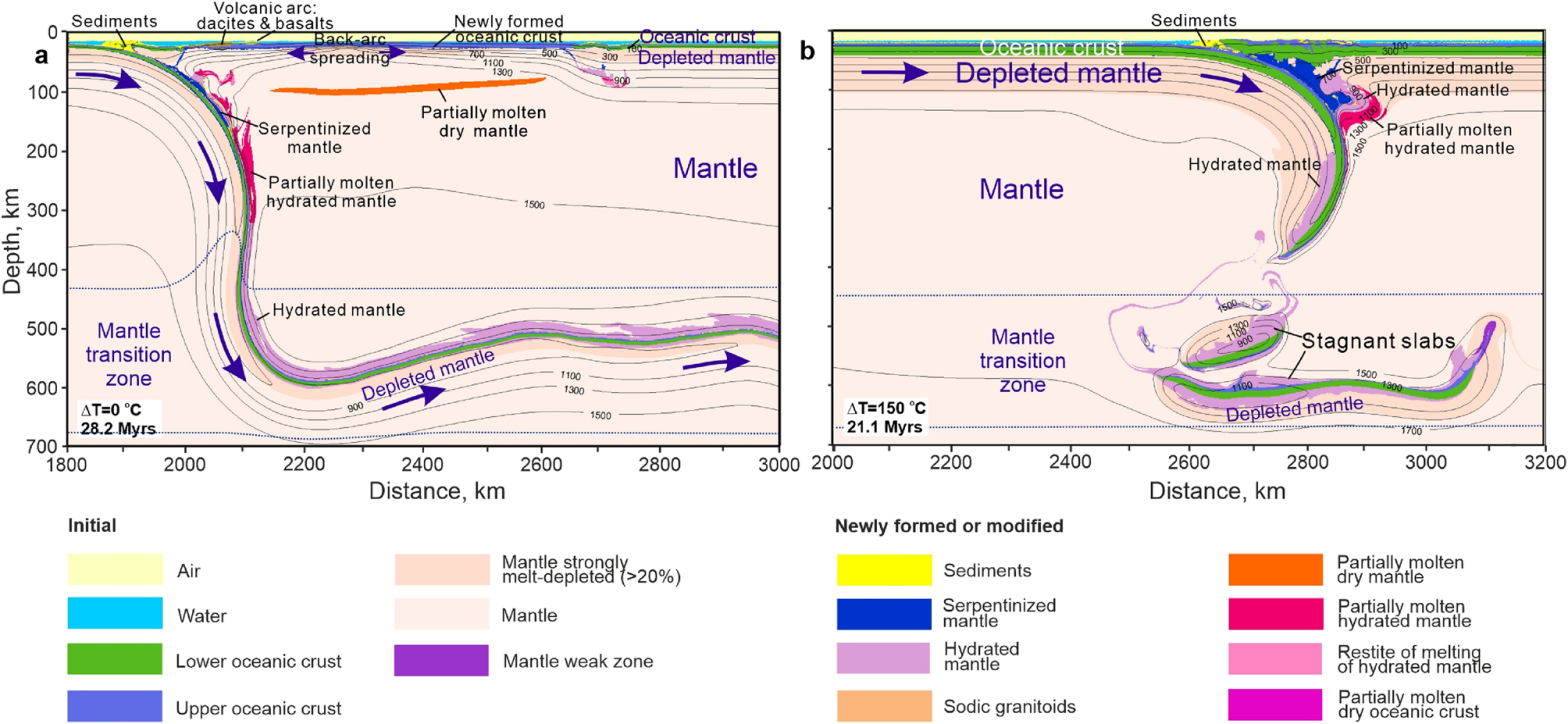
Subduction with relatively thin depleted mantle at constant convergence velocity of 5 cm/year. (**a**) Modern style of subduction with slab retreat and formation of island arcs with basaltic and dacitic volcanism, back-arc basins with a new oceanic crust at modern mantle temperature ΔT = 0 °C (T_p_ = 1300 °C). (**b**) One-sided subduction with voluminous serpentinization in the mantle wedge, dacitic and basaltic volcanism and elevated mantle temperature ΔT = 150 °C (T_p_ = 1450 °C). Dotted dark-blue lines indicate an actual upper and lower boundaries of the initial mantle transition zone. Arrows show direction of plate motion (not to scale) and viscous flow of the depleted mantle. The colour key is shown at the bottom of the figure. Prescribed velocity of the left-hand plate is 5 cm/year.

Subduction at modern mantle temperatures (∆T = 0 °C) occurs in a retreating mode, producing a magmatic arc with basaltic and dacitic volcanism and a large back-arc basin with a spreading center, where new oceanic crust and related depleted mantle are formed (Fig. 2a). The depleted mantle is relatively thin and descends with the slab into the Mantle Transition Zone (MTZ).

Subduction at ∆T = 150 °C and a convergence rate 5 cm/year is characterized by a strongly curved Z-shaped slab, rare slab break-off and the development of a relatively large subduction channel composed of serpentinites (Fig. 2b). Thickening of the depleted mantle of the slab (an embryonic viscous underplate, see below) develops at depths of 210–240 km in this numerical experiment.

**Figure 3:**
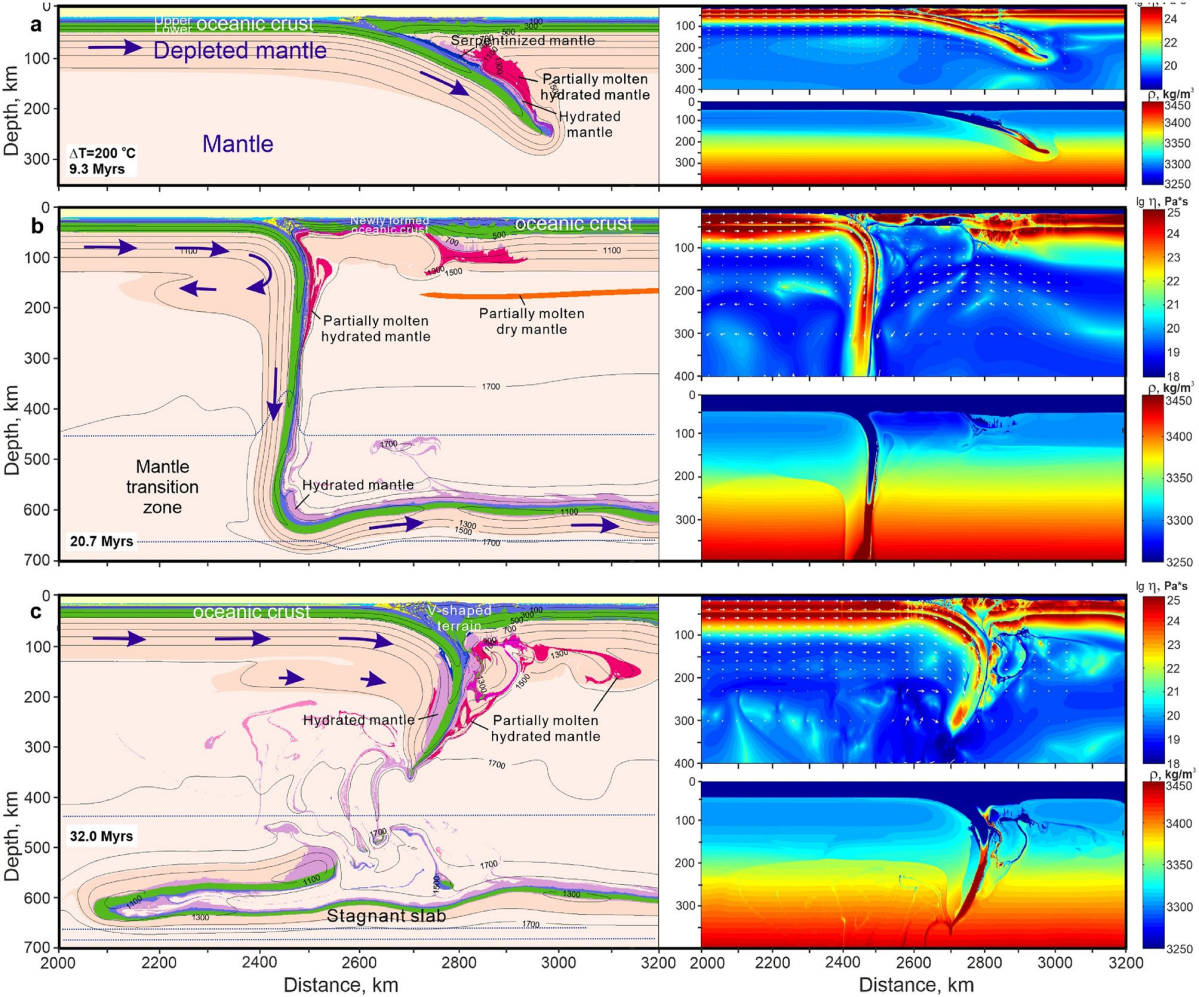
Development of viscous underplating during one-sided ocean–ocean subduction at constant convergence velocity of 5 cm/year and elevated mantle temperature ΔT = 200 °C (T_p_ = 1,500 °C). (**a**) Flat ocean–ocean subduction with highly depleted mantle with low density (9.3 myrs from the beginning of the experiment). Eclogitization of the subducting oceanic crust (density ≥ 3450 kg/m^3^) occurs at ~ 120 km depth. (**b**) Vertical subduction, development of the viscous underplate, slab retreat accompanied by back-arc spreading (20.7 myrs from the beginning of the experiment). Note the reverse viscous flow of the depleted mantle detached from the slab on the left-and formation of visous underplate. Eclogitization of the subducting oceanic crust (density ≥ 3450 kg/m^3^) occurs at ~ 250 km depth. (**c**) Formation of the V-shaped terrain during closure of the back-arc basin after slab break-off. Note that the depleted mantle is over-thickened around the subduction zone (32 myrs from the beginning of the experiment). Density and effective viscosity fields with velocity vectors are shown for each stage as separate panels at the bottom right and at the top right, respectively. Dotted dark-blue lines in b and c left column indicate an actual upper and lower boundaries of the mantle transition zone. Arrows show direction of plate motion (not to scale) and viscous flow of the depleted mantle. The colour key is as in Fig. 2. Prescribed velocity of the left-hand plate is 5 cm/year.

With one-sided subduction at ∆T = 200 °C and a convergence rate of 5 cm/year the slab remains relatively flat during the first 9 myrs (Fig. 3a); then it descends almost vertically to the bottom of the MTZ where it bends and flows horizontally. The main reason for slab stagnation at the MTZ is the negative Clapeyron slope of the perovskite phase transition and the deeper transition depth (pressure) in the oceanic crust compared to the mantle (“Method”). Subsequent evolution of the subduction zone involves slab retreat accompanied by formation of a back-arc basin (Fig. 3b) and followed by slab break-off, which leads to resumption of slab advance and closure of the back-arc basin (Fig. 3c). A V-shaped accretionary terrain with a thickened oceanic crust is formed at this stage. A peculiar feature of subduction with ∆T = 200 °C is the development of a pronounced viscous underplate—a layer of hot, ductile, positively buoyant, melt-depleted sublithospheric mantle detached from the downgoing slab (Fig. 3b,c), similar to the one developed under cratonic continental mantle in the oceanic-continental convergence models^17^. The viscous underplate is up to 60 km thick and 250 km wide, and separated from the overlying depleted mantle by ~ 10 km of nondepleted mantle.

### Two-sided subduction regime at ∆T = 250 °C

**Figure 4:**
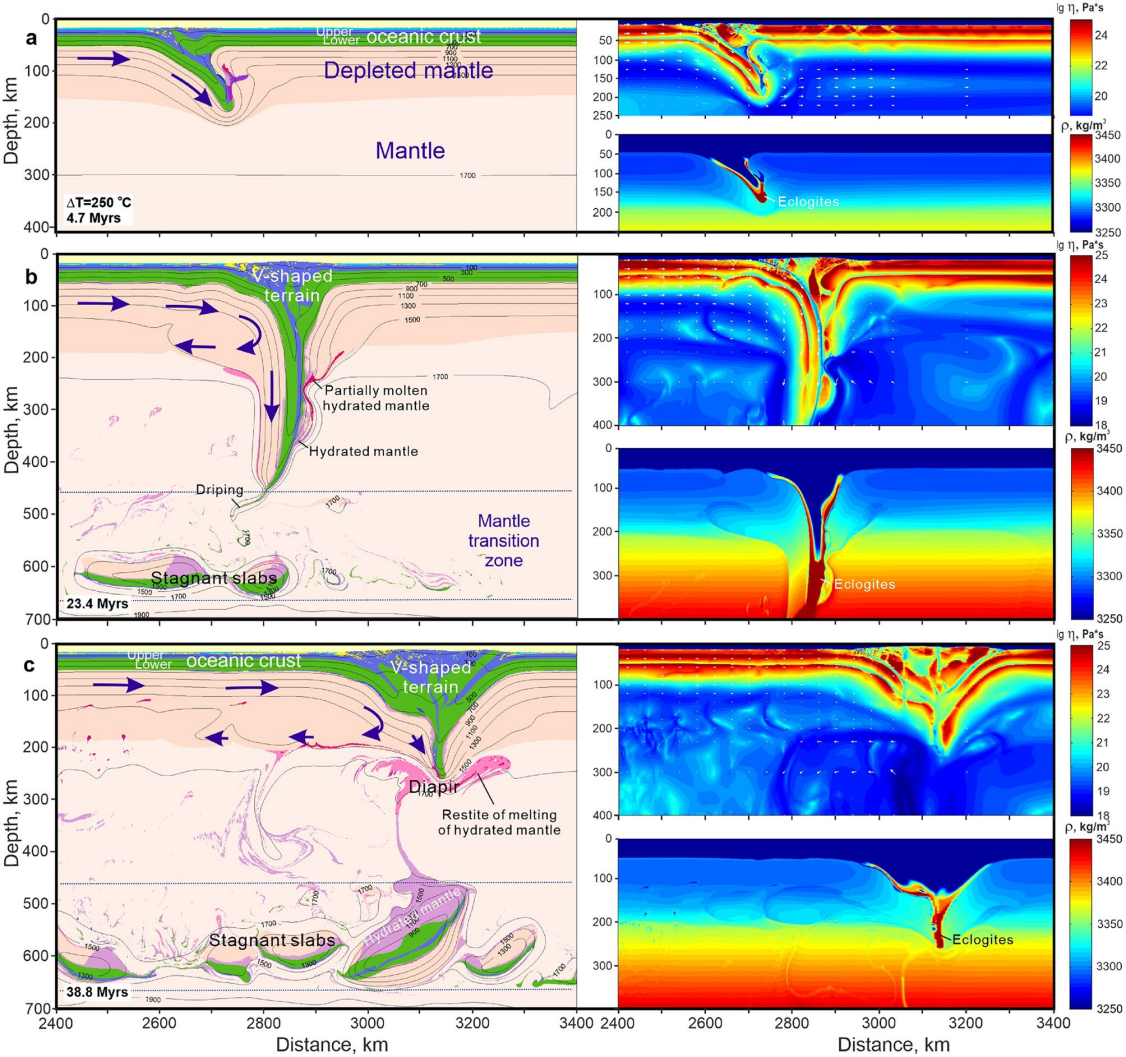
Formation of an accretionary V-shaped terrain and over-thickened melt-depleted mantle during two-sided subduction at constant convergence velocity of 5 cm/year and elevated mantle temperature ΔT = 250 °C (T_p_ = 1550 °C). (**a**) Flat ocean–ocean subduction with highly depleted mantle (4.7 myrs from the beginning of the experiment). Eclogitization of the subducting oceanic crust (density ≥ 3450 kg/m^3^) occurs at ~ 150 km depth. (**b**) Two-sided vertical subduction with V-shaped accretionary terrain atop over-thickened depleted mantle under the left-hand plate (23.4 Myr from the beginning of the experiment). Note the reverse viscous flow of the depleted mantle detached from the left-hand slab. Eclogitization of the subducting oceanic crust (density ≥ 3450 kg/m^3^) occurs at ~ 250 km depth. (**c**) Large V-shaped accretionary terrain and voluminous stagnant slabs in the mantle transition zone (38.8 Myr from the beginning of the experiment). Density and effective viscosity fields with velocity vectors are shown for each stage as separate panels at the bottom right and at the top right, respectively. Dotted dark-blue lines in b and c of the left column indicate upper and lower boundaries of the mantle transition zone. Arrows show direction of plate motion (not to scale) and viscous flow of the depleted mantle. The colour key is as in Fig. 2. Viscosity of the mantle is intrinsically dependent on pressure, temperature, degree of depletion and presence of fluid/melt (see “Method”). The rheological transition between the rigid lithospheric and low-viscosity asthenospheric (sublithospheric) mantle approximately corresponds to the 1300 °C isotherm. Prescribed velocity of the left-hand plate is 5 cm/year.

When two oceanic plates converge at a constant velocity (5 cm/year) the initially prescribed subduction of one of the plates (one-sided subduction) is shortly followed by a steep downwelling of both plates in a nearly symmetrical convergence zone, i.e. two-sided subduction with trench advance (Fig. 4). Compressive shortening of the crust produces a V-shaped (in 2-D section) terrain of overthickened crust with a wide accretionary wedge above and a deep crustal column consisting of doubled oceanic crust and melt-depleted lithospheric mantle (Fig. 4). The terrain’s crust becomes about 100 km thick and 300 km wide over 38 myrs of convergence (Fig. 4c). The accretionary wedge comprises upper oceanic crust (basalts + sediments) and detached blocks of the lower crust (gabbro and related rocks). The double lithospheric column descends almost vertically down to the MTZ, and subsequently experiences frequent break-off (Fig. 4b,c). The mantle wedge and magmatic arc characteristic of modern-style one-sided subduction are not developed during this double-sided subduction.

The depleted-mantle layers inside the subduction column are thinner than those that entered the two-sided subduction zone (Fig. 4b). This reflects detachment of the ductile, positively buoyant, melt-depleted sublithospheric mantle from the downgoing slab^17^. The detached mantle flows viscously in the opposite direction from the slab (Fig. 4b), which leads to thickning of the melt-depleted mantle under the oceanic crust by up to 40% (cf. Fig. 4a,c).

**Figure 5:**
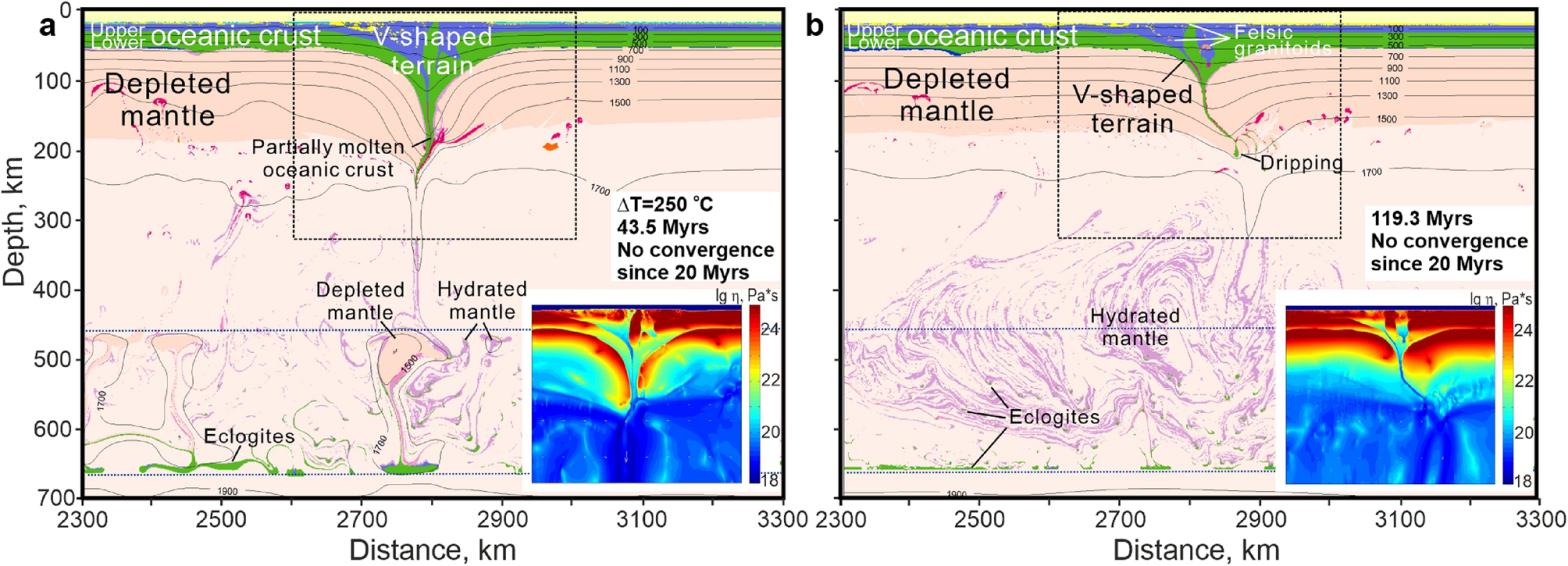
Shrinking of the V-shaped terrain and formation of sodic granitoids after stop pushing the plate at 20 myrs and elevated mantle temperature ΔT = 250 °C (T_p_ = 1550 °C). (**a**) V-shaped terrain reaches ~ 200 km depth. Gravitational separation of the slab-related depleted mantle and eclogites in the transition zone (at 43.5 myrs from the beginning of the experiment). (**b**) Shrinking of V-shape terrain down to ~ 100 km depth. Formation of sodic granites derived from the metabasic rocks. Stretching of the hydrated peridotites with rare eclogites in the transition zone and in upper mantle (at 119.3 myrs from the beginning of the experiment). The insets show effective viscosity fields and stiffening of the V-shape terrain in time.

Since subduction in the early Earth is often considered as an episodic process^14^ we investigated the evolution of the thick V-shaped terrain in a subsequent non-subduction regime by switching off convergence at 19 myrs, when the root had almost reached the MTZ (cf. Fig. 4b). At the early stage, the deep part of the terrain breaks off and sinks into the MTZ, where dense eclogites and more buoyant hydrated depleted peridotites start to separate into different levels of the MTZ (Fig. 5). Subsequent thermal relaxation leads to (1) the drip of the eclogitized upper crust (former basalts) of the terrain sometimes together with fragments of depleted lithospheric mantle; (2) rheological weakening of the deep portions of the terrain (Fig. 5a inset); (3) partial melting of the deep-seated crustal rocks (eclogites) to produce sodic granitoids (TTGs), typical of the continental crust (Fig. 5b). The descending eclogites hydrate the ambient mantle, which stretches out into a complex mixing pattern consisting of numerous thin, buckling layers of hydrated mantle in the dry mantle (Fig. 5b). The dripped eclogites are stored at the bottom of the MTZ where they mix with the earlier stagnant-slab eclogites (Fig. 5). The V-shape terrains are preserved long after the termination of convergence. According to Fig. 5a the maximal thickness of the eclogitized crustal wedge in the terrain can reach up to ~ 180 km. However, this maximal thickness is a transient feature, which will not be preserved in its original shape and size in the geological history (cf. Fig. 5a,b).

### Effect of convergence rate

We tested the effect of this parameter on the subduction regime by numerical experiments at plate velocities of 2 and 10 cm/year. These experiments generally reproduce key features (1-sided subduction at ∆T ≤ 200 °C vs a 2-sided regime at ∆T = 250 °C, formation of overthickened melt-depleted mantle at ∆T = 200–250 °C) of the runs with 5 cm/year convergence rate (Supplementary Figs. S2 and S3). However, there are some important differences.

For example, faster convergence (10 cm/year) at ∆T = 250 °C creates several coexisting double-sided subduction zones that build V-shaped accretionary terrains (Supplementary Fig. S2a). It is accompanied by frequent slab break-off and accumulation of the stagnant slabs in the MTZ. Subduction at 10 cm/year at mantle temperatures ∆T = 200, 150 and 0 °C produces one-sided subduction regimes that form Z-shaped slabs (more or less deformed near the MTZ). No slab retreat occurs at this velocity. Over-thickened depleted mantle is formed only at ∆T = 200 °C and 150 °C by stagnation of the depleted ductile sublithospheric mantle under the horizontal portion of the subducting plate (Supplementary Fig. S2b,c).

Convergence at 2 cm/year results in dripping of the eclogitized oceanic crust from the bottom of the V-shaped terrain to the bottom of the MTZ during two-sided subduction at ∆T = 250 °C (Supplementary Fig. S3a). Significant stretching of the slabs as well as large serpentinite-dominated subduction channels are typical of one-sided subduction regimes at ∆T = 200 and 150 °C (Supplementary Fig. S3b,c). Subduction at ∆T = 200 °C leads to the development of the viscous underplate.

### Accumulation of melt-depleted peridotites in the upper mantle

**Figure 6:**
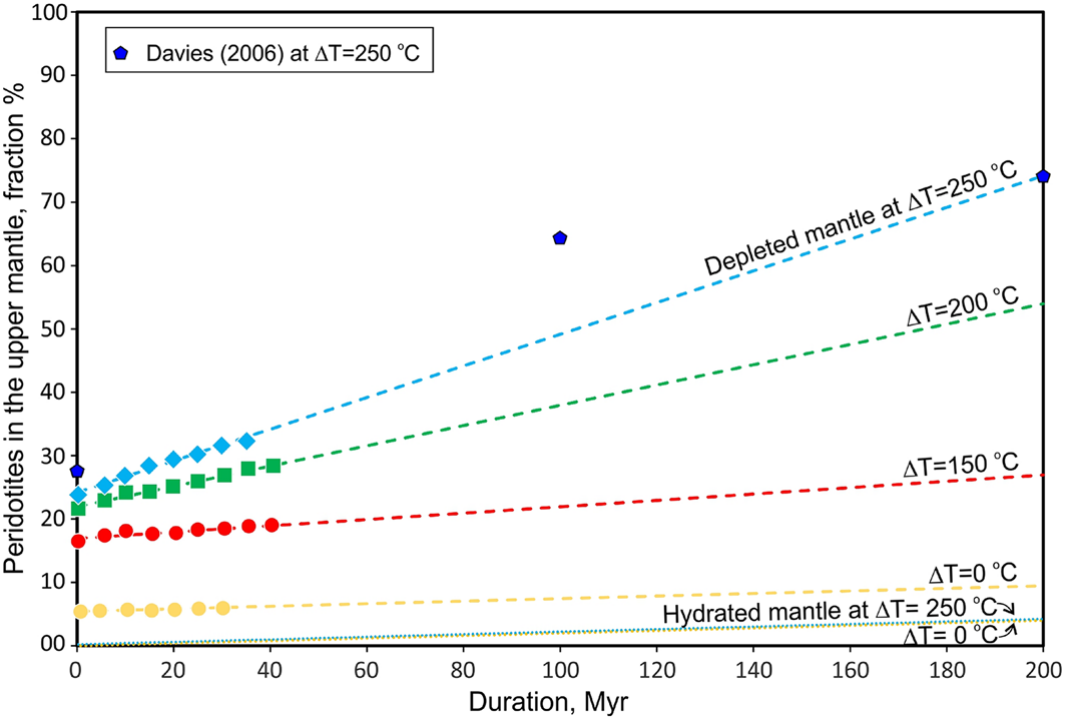
Accumulation of depleted (> 20%) peridotites in the upper mantle at different potential mantle temperatures ΔT = 0–250 °C. Filled circles, quadrangles and diamonds are results of our numerical experiments, related dashed lines—linear fit to the points from the numerical model results. Blue pentahedrons show the modeling results by Davies^26^ at ∆T = 0–250 °C.

Since asthenospheric depleted mantle detaches from the slabs (Figs. 3 and 4) and does not enter the MTZ, it accumulates gradually in the upper mantle. The accumulation rate was quantified by estimating the volume fraction of depleted peridotites (melt depletion > 20%) in the upper mantle in the numerical experiments for different values of ∆T (Fig. 6). The higher the mantle temperature (which leads to thinner lithosphere and thicker depleted asthenospheric peridotites, Fig. 1), the more rapidly buoyant, depleted peridotites accumulate in the upper mantle. Extrapolation of the linearly growing volume fraction to 200 million years of subduction predicts the replacement of 55 and 80% of the upper mantle by depleted peridotites at ∆T = 200 oC and ∆T = 250 °C, respectively (Fig. 6). In contrast, the fraction of depleted peridotites in the upper mantle during subduction at ∆T = 150 °C increases very slowly and becomes almost constant at ∆T = 0 °C. In the latter case it is controlled exclusively by decompression melting in the back-arc spreading zone, whereas melt-depleted peridotites subduct with the slab without storage or/and detachment (Fig. 2a). These effects are mainly related to the deeper initiation of decompression melting and the correspondingly higher degree of melting and depletion of the ambient oceanic asthenosphere rising under mid-ocean ridges^3^ at higher mantle temperature (Fig. 1), where larger volumes of more depleted mantle form at mid-ocean ridges and become mobilized by subduction.

The fraction of hydrated peridotites that has been affected by fluids released from the downgoing slab (i.e. metasomatically modified peridotites in the upper mantle) remains relatively small, independent of mantle temperature, and increases very slowly with time (Fig. 6). Owing to that, metasomatic alteration of upper mantle peridotites by fluids does not promote recycling of a glut of depleted peridotites (fertilized depleted peridotite^27^) in the hot Archean mantle.

## Discussion

It is often suggested that Archean geodynamics was dominated by plume tectonics and lithospheric delamination (dripping-off) processes, because rheologically weak lithosphere was produced by infiltration of partial melts derived from the asthenosphere at high mantle temperatures^15,19,21,22^. However, numerical modeling demonstrates that if extensional tectonics operated in the Archean, refractory depleted mantle effectively stiffens oceanic plates and they become able to subduct under imposed convergence (Figs. 3 and 4). Motion of the plates may be initiated by plumes^25^ and sustained by convective drag and slab pull. Eclogitization takes place at depths > 200 km in our models (Figs. 3 and 4); this clearly corresponds to ultrahigh pressure (UHP) metamorphic conditions, which are typically attributed to modern plate tectonics. As suggested by van Hunen and van den Berg^28^, UHP rocks probably are lacking in ancient metamorphic terrains because of inefficient exhumation and frequent slab breakoff. The models also indicate the problem with the efficiency of the eclogite-driven slab-pull mechanism as a driver of continued subduction, since the lower part of the slabs containing thick eclogitized oceanic crust repeatedly breaks off, thereby reducing the slab pull in both one-sided and two-sided subduction.

Two-sided subduction requires a low plate strength^29^, which is attained in the numerical models at Archean mantle temperatures ∆T = 250 °C (Fig. 4), and ∆T = 260 °C^11^. The weakness of oceanic lithosphere in the subduction zone mainly results from the thick mafic crust becoming ductile at the higher oceanic Moho temperatures and partly decoupling from the lithospheric mantle. We suggest that three features of symmetrical two-sided subduction (Figs. 4 and 5) deserve further attention and investigation: (1) the double sources of metasomatic fluids, (2) the lack of a mantle wedge, which is always develops above an obliquely-subducting slab and (3) lack of the modern-style magmatic arc, which needs one-sided rather than double-sided subduction^29^. These features predict a larger reservoir of subducting metasomatic fluids than in one-sided subduction, and subvertical ascent of the released fluids through the over-thickened V-shaped terrain, thus hydrating the rocks by metamorphic reactions. In our models, this upward fluid flow is clearly evidenced by the water loss from the deeply subducted crustal rocks of the doubled slab and the lack of significant hydration in the adjacent mantle peridotites (Supplementary Fig. S4). In this regime, subduction could not create a shallow hydrous mantle reservoir as happens in the mantle wedges of modern subduction on Earth^30,31^. Phase-equilibrium modeling of mineral assemblages in subducting oceanic crust demonstrates^32^ that if the oceanic crust that formed on the hot, early Earth was rich in magnesium oxide (MgO), greenschist-like metamorphic rocks (formed today at low temperatures and pressures) would have formed in subduction zones instead of blueschists (high pressure-low temperature rocks formed in modern subduction zones). These ancient metamorphic rocks have higher contents of water than younger blueschists^32^, implying that V-shaped terrains might be an important hydrologic reservoir in the early Earth.

Although our melting results do not demonstrate the formation of sodic granites (TTGs) by partial melting of the downgoing oceanic crust, we can predict that V-shaped terrains consisting of metamorphic and magmatic rocks of with oceanic-crust protoliths might evolve into domains with TTGs; i.e. development the embryonic continental crust. This would occur by partial melting of thickened metabasic rocks during the thermal relaxation of the V-shaped terrain after the termination of two-sided subduction (Fig. 5b).

The ancient nuclei of continental crust (cratons) formed in the Archean are underlain by overthickened lithospheric mantle (mantle keels) composed of strongly depleted peridotites of similar age^33^. The depleted peridotites from the keels have not been used in our experiments to constrain Precambrian magmatism and potential mantle temperatures; fertile (least depleted) peridotites^34^, which are closer to the composition of the global geochemical reservoir known as Primitive mantle (or pyrolite)^35^ have been preferred. This is consistent with the relatively small amount of depleted peridotites produced by partial melting even during Archean melt extraction at ∆T = 250 °C (Fig. 1). However, our new numerical models demonstrate that depleted mantle, once produced in Archean extensional tectonic settings (e.g. spreading ridges) is not easily recycled in subduction zones but accumulates in the upper mantle via development of viscous underplates or stagnation of the depleted peridotites under horizontal portions of subducting plates (Fig. 4; Suppl. Fig. S2b,c). Extrapolation of these data to 200 myrs of subduction suggests substantial replacement of the upper mantle by depleted peridotites at ∆T = 200–250 °C (Fig. 6). This is consistent with 2-D numerical models of subduction with kinematically prescribed motion of the oceanic plates at mantle temperatures of 1550 °C for 500 myrs^26^. By counting tracers that represent the mafic component of the mantle composition, we find that the maximum content of depleted peridotites in the upper mantle is reached at 200 myrs^26^, which coincides with the upper limit of our extrapolation (Fig. 6). These results support a geochemical argument^36^ that the depletion of the upper mantle is related more to the extraction of mafic material, rather than of continental crust. According to the modeling results of^17^, the depleted peridotites that built the mantle keels under the cratons are of oceanic affinity and their complementary oceanic crust rocks were recycled into the MTZ via the subduction zone. Accordingly, the geochemical record of the magmatism related to the development of the current convecting upper mantle is restricted or biased^37^.

Predictions from our models may contribute to better understanding of the formation of cratonic mantle keels, recently addressed in several papers^17,18,38^. Indeed, if large fractions of the early Earth upper mantle are composed of the depleted peridotites, then the enigmatic mechanism of keel formation is transformed into the problem of (thermal) accretion of the available depleted mantle to the keels and their preservation (longevity) through geological time^39,40^ as well as recycling of non-accreted depleted upper mantle by mantle convection. The observed secular decrease in the thicknesses of mantle keels^41^ correlates with our predicted decreasing rates of depleted-mantle addition with decreasing mantle temperature (Fig. 6). This correlation may also suggest that the depleted asthenospheric mantle (i.e., not accreted to cratonic keels) can be efficiently mobilized and recycled into and mixed with the lower mantle by thermal-chemical whole-mantle convection^42^.

## Method

The regional 2D magmatic-thermomechanical model simulates the subduction of an oceanic plate under another oceanic plate (intraoceanic subduction). The governing equations of conservation of momentum, mass and energy are solved with the use of the code I2VIS^43^, based on conservative finite differences and a non-diffusive marker-in-cell technique applied on a staggered non-uniform Eulerian grid. The 4000 × 1000 km numerical model domain is resolved with a non-uniform 2041 × 381 rectangular grid that provides the highest grid resolution of 1 km in the 1500-km-wide and 200-km-thick subduction area of the model. More than 70 million Lagrangian markers are used to carry material properties, temperature, water content and melt-depletion, which are initially distributed over the very dense randomly-perturbed Lagrangian grid. The model includes creeping flow with thermal and chemical buoyancy forces; it accounts for phase transitions in the mantle and basaltic and gabbroic crust that is described below the eclogite, olivine-wadsleyite and ringwoodite-postspinel phase transitions, and considers the effects of adiabatic, shear, latent and radioactive heating. In addition, phase equilibria for basalt/gabbro of MORB composition and peridotite of pyrolite composition are taken into account^44–46^. Complete details of this method, allowing for its reproduction, are provided elsewhere^43^.

Numerical experiments were carried out using an approach similar the one presented in our recent paper^17^. In this approach, we did not stratify the mantle into lithosphere and asthenosphere; we instead introduced a melt-depleted mantle layer that was formed in the spreading ridge due to decompression melting and extraction of the melt to form oceanic crust. Lithosphere and asthenosphere were established intrinsically according to the rheology of olivine as a function of pressure and temperature; the transition usually corresponds to the 1300 °C isotherm^47^. Partial melting of peridotite as parametrized by Katz et al.^48^ shows that melt-depleted mantle beneath the oceanic crust is located within the lithospheric mantle until the mantle potential temperature *T*_p_ = 1400 °C is reached (Phanerozoic and Neoproterozoic). At higher mantle temperatures it significantly exceeds the thickness of the lithospheric mantle (Fig. 1) thus representing an important reservoir of positively buoyant, refractory and dry mantle, which is of key interest for this paper as well.

We conducted a series of numerical experiments with different mantle potential temperatures (*T*_p_) of 1300, 1450, 1500 and 1550 °C (i.e., ∆*T* = 0, 150, 200 and 250 °C hotter than the present-day values, reaching temperatures representative of Precambrian conditions^1,2,4,34^, and prescribed constant subducting plate velocities of 2, 5, and 10 cm/year (Supplementary Table S1). The prescribed horizontal plate velocity is imposed in the grid nodes located between 100 and 1800 km horizontally and between the oceanic Moho and the 1300 °C isotherm vertically. Our previous study^20^ demonstrated that, due to the low asthenosphere viscosity at elevated mantle potential temperature in the Archean, characteristic subduction velocity increases strongly and our prescribed elevated subduction velocities of 10 cm/year are thus plausible. In some models, this prescribed plate velocity condition was deactivated at 20 myrs and the subducting plate is allowed to move freely (Suppl. Table S1). The initiation of subduction is enabled by prescribing an initial weak zone (inclination angle 20°) with the rheology of wet olivine^49^ and low brittle/plastic strength (Supplementary Table S2).

We assumed that modern oceanic crust is 7 km thick and consists of a layer of hydrothermally altered basalts (2 km thick) underlain by layer of gabbroic rocks (5 km thick) with the rheology of wet quartzite and plagioclase^49^, respectively (Supplementary Table S2). In a series of experiments related to Precambrian subduction, the thickness of the oceanic crust was increased linearly from 20 to 30 km with mantle potential temperature *T*_p_ increasing from 1450 to 1550 °C (Supplementary Table S1). The mantle is represented by anhydrous peridotite, which is initially subjected to depth-dependent melt-depletion in accordance with the mantle potential temperature using the melting model^48^.

All mechanical boundary conditions are free-slip at all boundaries. The top surface of the lithosphere is treated as an internal free surface^50^. This upper boundary evolves by erosion and sedimentation according to a Eulerian transport equation^19,51^. The surface slope, φ_max,_ for the accumulated sedimentary prism varies up to 35° (tgφ_max_ = 0.7).

The density of rocks varies with pressure (*P*) and temperature (*T*) according to the equation

$$\uprho _{{{\text{P}},{\text{T}}}} = \uprho 0 \cdot \left[ {1 - \upalpha \left( {T - T0} \right) \cdot 1 + \upbeta \left( {P - P_{0} } \right)} \right],$$

where ρ_0_ is the standard density at *P*_0_ = 1 MPa and *T*_0_ = 298 K, and α and β are the coefficients of thermal expansion and compressibility, respectively (Supplementary Table S2).

Variations of the densities of peridotites and metabasalts/metagabbro—key lithologies that strongly affect kinematics and style of subduction—were treated according to Mishin et al.^44^.

Our model takes into account the transformations of olivine into wadsleyite and ringwoodite (spinel transition)^45^ and then into bridgmanite (perovskite transition) in the mantle^46,52^. In basaltic and gabbroic crust, we take into account density changes due to eclogitization and formation of stishovite and perovskite^44^. Eclogitization of subducted basaltic and gabbroic crust is taken into account by linearly increasing the density of the crust with pressure from 0 to 16% in the *P–T* region between the experimentally-determined garnet-in and plagioclase-out phase transitions in basalt^53^. The perovskite transition in the crust is prescribed with the same Clapeyron slope but at 5 GPa higher pressure than in the mantle^44^. The physical parameters for each experiment are presented in Supplementary Table S2.

During the running of the model, water is liberated from the subducted oceanic crust as a consequence of dehydration reactions and compaction^19^. We assume incomplete hydration of the mantle wedge as a consequence of the channelization of slab-derived fluids. To account for this behavior, we arbitrarily assign 2 wt% H_2_O as an upper limit for mantle wedge hydration. The hydrated mantle is subdivided into two parts (1) an upper, colder serpentinized zone, and (2) a lower, warmer and hydrated, but not serpentinized zone. The stable mineralogical composition and water content were obtained by free energy minimization^54^ as a function of pressure and temperature from thermodynamic data^55^. Due to existing uncertainties around the high-pressure mineralogy of subducted crust and hydrated mantle in the mantle transition zone and the amount of water in the nominally anhydrous minerals, we cannot predict very accurately the amount of water that is released from subducting slabs in the mantle transition zone. However, this amount is high enough to trigger hydrous diapirism processes in the mantle transition zone^56,57^ that are observed in our models.

Melting of the mantle and the crust, as well as melt extraction and percolation across the crust-mantle interface and to the surface are implemented in a simplified manner^58^. According to our model, magma added to the crust is balanced by melt production and extraction in the source mantle/crustal region. Melt extracted from the mantle rises and is added to the crust as hot intrusions (plutons, 70% of all melts) and to the surface as volcanic rocks (30% of all melts)^16,58^.

One key component of our numerical model is that it takes into account the decrease in the mantle density related to melt extraction. The standard density of the melt-depleted mantle is corrected for the degree of depletion as^25^:

$$\uprho _{{0\left( {{\text{depl}}} \right)}} = \uprho _{0} \left( {1 - 0.04\sum _{{\text{m}}} M_{{{\text{ext}}}} } \right),$$

where ρ_0(depl)_ is the standard density of depleted solid mantle and Σ_m_*M*_ext_ is the degree of melt extraction, which changes with time.

The volumetric degree of melting *M*_0_ in partially molten rocks is computed similarly to^58^. For the mantle, we use the *P*–*T*–H_2_O-dependent melting model for peridotite^48^. For crustal rocks, we assume that the degree of both hydrous and dry melting is a piecewise-linear function of *P*–*T*^43^,

$$M_{0} = \left\{ {\begin{array}{*{20}l} 0 \hfill & {T <T_{{solidus}} } \hfill \\ {\frac{{T - T_{{solidus}} }}{{T_{{liquidus}} - T_{{solidus}} }}} \hfill &{T_{{solidus}} <T <T_{{liquidus}} } \hfill \\ 1 \hfill &{T >T_{{liquidus}} } \hfill \\ \end{array} }\!\! ,\right.$$

where *T*_solidus_ and *T*_liquidus_ are, respectively, the solidus temperature and dry liquidus temperature at a given pressure and rock composition (Supplementary Table S2).

The effect of latent heat due to equilibrium melting/crystallization is included implicitly by increasing the effective heat capacity (*C*_P,eff_) and thermal expansion coefficient (α_eff_) of the partially molten/solidified material^59^,

$$\begin{aligned} & C_{{{\text{P}},{\text{eff}}}} = C_{{\text{P}}} + H_{{\text{L}}} \left( {\frac{{\partial M}}{{\partial T}}} \right)_{{{\text{P}} = {\text{const}}}} ,\;{\text{and}} \\ & \upalpha _{{{\text{eff}}}} = \upalpha + \uprho \frac{{H_{{\text{L}}} }}{{\text{T}}}\left( {\frac{{\partial M}}{{\partial P}}} \right)_{{{\text{T = const}}}} \\ \end{aligned}$$

where *C*_P_ is the heat capacity and *H*_L_ is the latent heat of melting.

The viscosity for dislocation creep depends on strain rate, pressure and temperature and is defined in terms of deformation invariants^49^ as follows,

$${\upeta }_{\text{creep}}={\left({\dot{\upvarepsilon }}_{\text{II}}\right)}^{(1-n)/n}{A}_{\text{D}}^{-1/n}\text{exp}\left(\frac{E+VP}{nRT}\right),$$

where ${\dot{\upvarepsilon }}_{\text{II}}=\sqrt{1/2{\dot{\upvarepsilon }}_{\text{ij}}{\dot{\upvarepsilon }}_{\text{ij}}}$ is the square root of the second invariant of the strain rate tensor ${\dot{\upvarepsilon }}_{\text{ij}}$ and *A*_D_, *E, V* and *n* are the experimentally determined flow law parameters: the material constant, the activation energy, the activation volume and the stress exponent, respectively (Supplementary Table S2).

The Drucker–Prager yield criterion^49^ is implemented by limiting creep viscosity as follows,

$${\upeta }_{\text{creep}}\le \frac{c+P\gamma }{2{\dot{\upvarepsilon }}_{\text{II}}},$$

where *c* is the compressive strength at *P* = 0, and γ is the effective internal friction coefficient, which includes the effects of fluid and melt weakening,

$$\begin{aligned} & \upgamma = \upgamma _{{{\text{dry}}}} \uplambda _{{{\text{fluid}}}} \;{\text{and}}\;\upgamma = \upgamma _{{{\text{dry}}}} \uplambda _{{{\text{melt}}}} ,\;{\text{and}}\;\upgamma _{{{\text{dry}}}} {\text{-internal}}\;{\text{friction}}\;{\text{coefficient}}\;{\text{of}}\;{\text{dry}}\;{\text{rocks}}, \\ & \uplambda _{{{\text{fluid}}}} = 1 - \frac{{P_{{{\text{fluid}}}} }}{{P_{{{\text{solid}}}} }},\;\uplambda _{{{\text{melt}}}} = 1 - \frac{{P_{{{\text{melt}}}} }}{{P_{{{\text{solid}}}} }}. \\ \end{aligned}$$

According to this rheological model, the pore fluid pressure *P*_fluid_ and melt pressure *P*_melt_ reduce the yield strength σ_yield_ of fluid/melt-bearing rocks^60^. As established in our previous study^20^, values of λ_fluid_ = 0.1 and λ_melt_ = 0.01 provide realistic volumes and compositions of arc volcanic rocks for contemporary intraplate subduction. Our rheological model also accounts for slab weakening at depths greater than 200 km^61^ by gradually decreasing the upper cutoff viscosity value from 10^25^ to 10^22^ Pa s in the depth interval between 200 and 400 km. No viscosity increase has been applied at the perovskite transition in the mantle.

## Supplementary Information

Supplementary Information.
