# Supplementary material for: Depletion of the upper mantle by convergent tectonics in the Early Earth
Source: Sci Rep. 2021 Nov 2;11:21489. doi: 10.1038/s41598-021-00837-y (PMC8563749; doi:10.1038/s41598-021-00837-y)
Supplement: Supplementary file 1 — Supplementary Information. [file 41598_2021_837_MOESM1_ESM.docx]

**Depletion of the upper mantle by convergent tectonics in the Early Earth**

A.L. Perchuk, T.V. Gerya, V.S. Zakharov, W.L. Griffin

**SUPPLEMENTARY DATA**

**
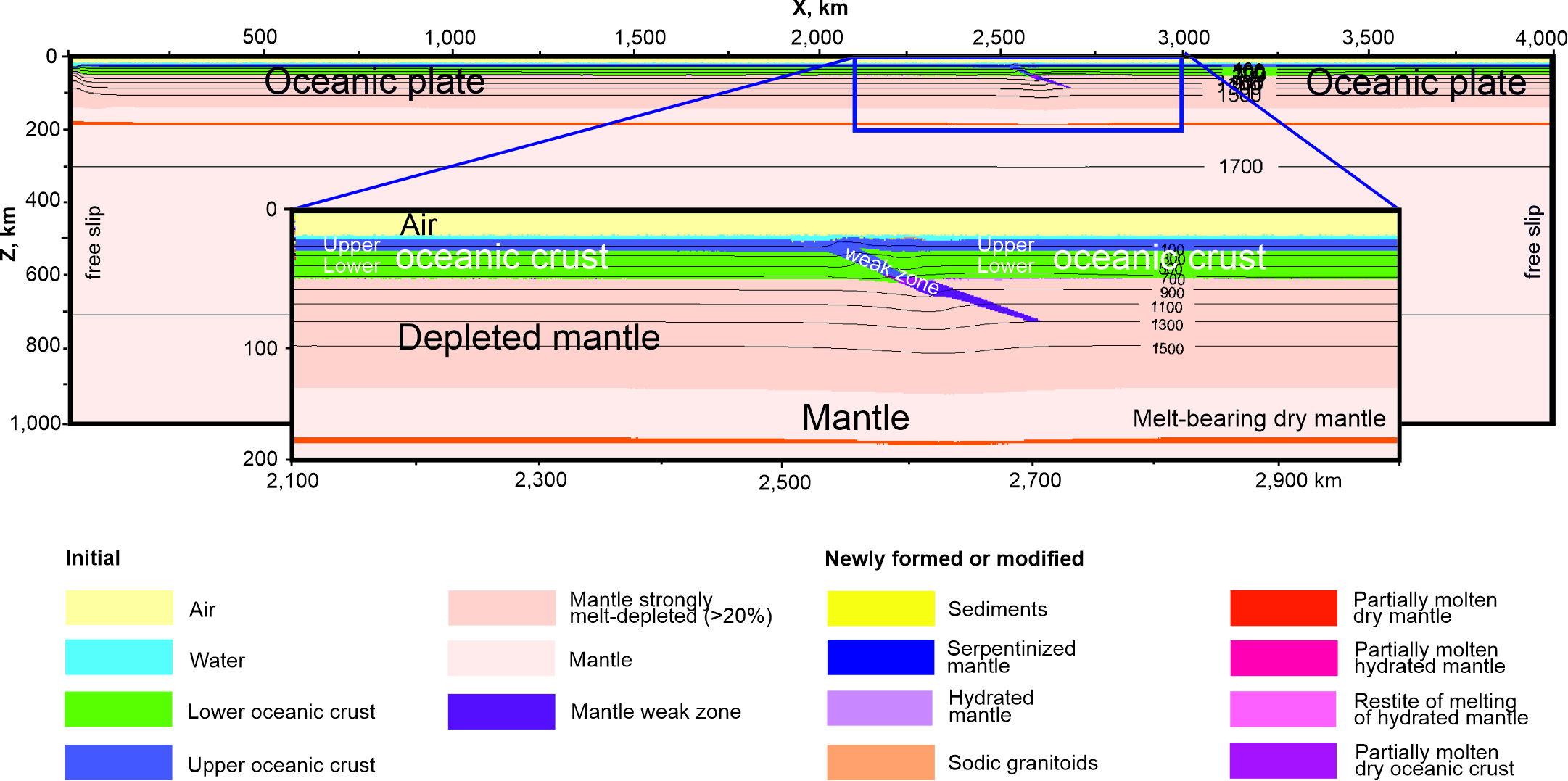
**

**Suppl. Fig. S1**. Design and boundary conditions of the numerical model. Black lines are isotherms at increments of 200 °C starting from 100 °C. Colours indicate materials (for example, rock type or melt). Mantle with a degree of melt-depletion of more than 20% is shown in peach. Model parameters are for elevated mantle potential temperature (T_p_) of 1550 °C (ΔT = 250 °C). The zoomed-in area shows the prescribed incipient subduction zones. The colour key for different materials is shown at the bottom.


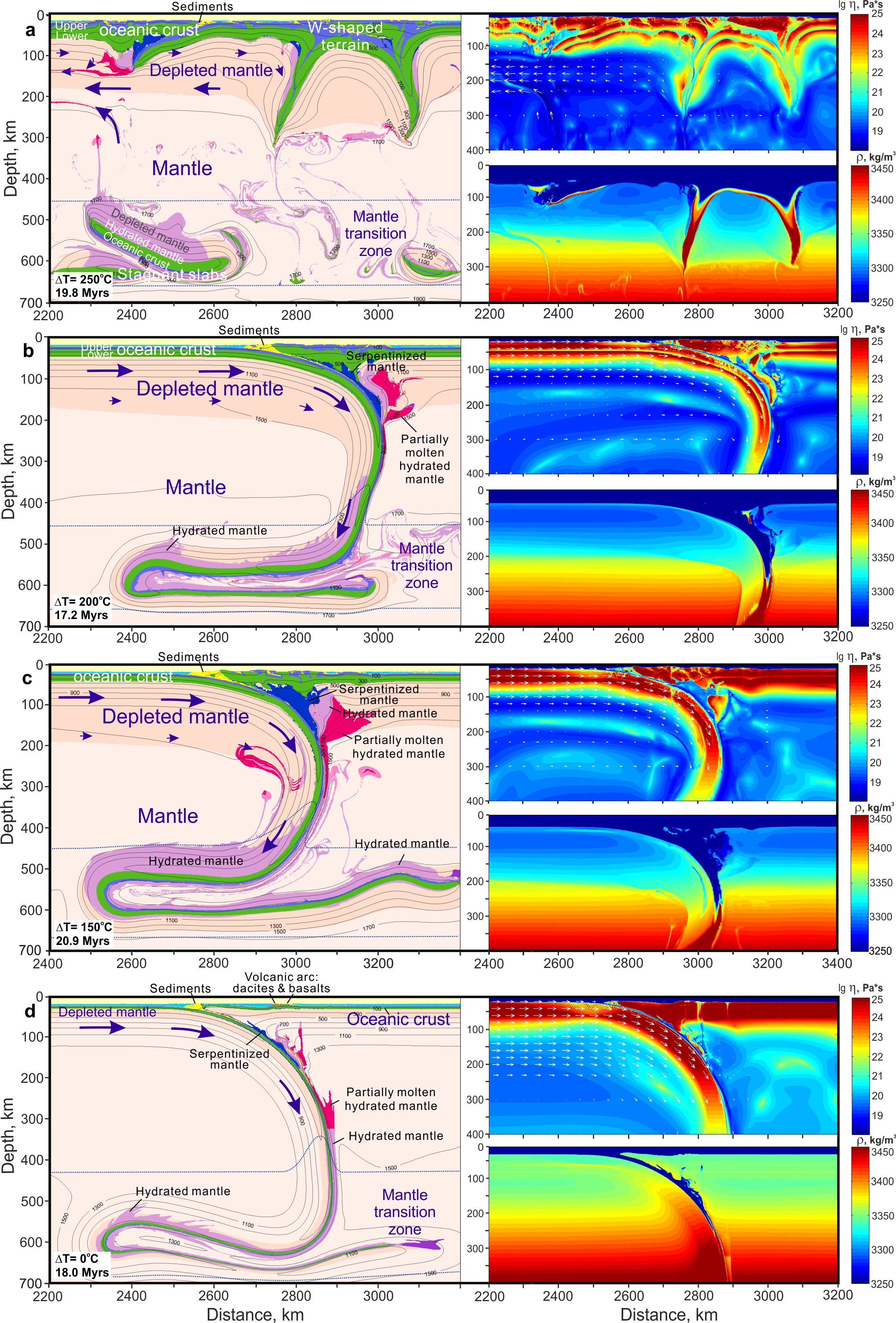


**Suppl. Fig. S2.** Changes in subduction styles at different potential mantle temperatures and velocity rate 10 cm/yr. a, Two-sided subduction leads to formation of over-thickened depleted mantle and V-shaped terrains (grouped in a W-shaped terrain) at elevated mantle potential temperature (T_p_ = 1550 °C; ΔT = 250 °C; 19.8 myrs from the beginning of the experiment). b, Z-shaped one-sided subduction with formation of over-thickened depleted mantle under the subducting plate and accretionary terrain in the overriding plate at elevated mantle potential temperature (T_p_) of 1500 °C (ΔT = 200 °C;17.2 myrs from the beginning of the experiment). Note that the overthickened depleted mantle is formed owing to rapid reduction of the velocity in lower part of the viscous depleted-peridotite layer. c, Z-shaped subduction with formation of over-thickened depleted mantle under the subducting plate and a serpentinized subduction channel in the hanging wall mantle at elevated mantle potential temperature (T_p_ = 1450 °C; ΔT = 150 °C; 20.9 myrs from the beginning of the experiment). Note that over-thickened depleted mantle is also formed owing to rapid reduction of the viscous depleted peridotites. d, Z-shaped subduction with formation of island arcs with basaltic and dacitic volcanism at modern mantle potential temperature (T_p_ = 1300 °C; ΔT = 0 °C; 20.9 myrs from the beginning of the experiment). Density and effective-viscosity fields with velocity vectors are shown for each stage as separate panels at the bottom right and at the top right, respectively. Dotted dark-blue lines in the left column indicate upper and lower boundaries of the MTZ. Arrows show direction of plate motion and viscous flow of the depleted mantle. The colour key is as in Fig. 2 and Supplementary Figure S1.


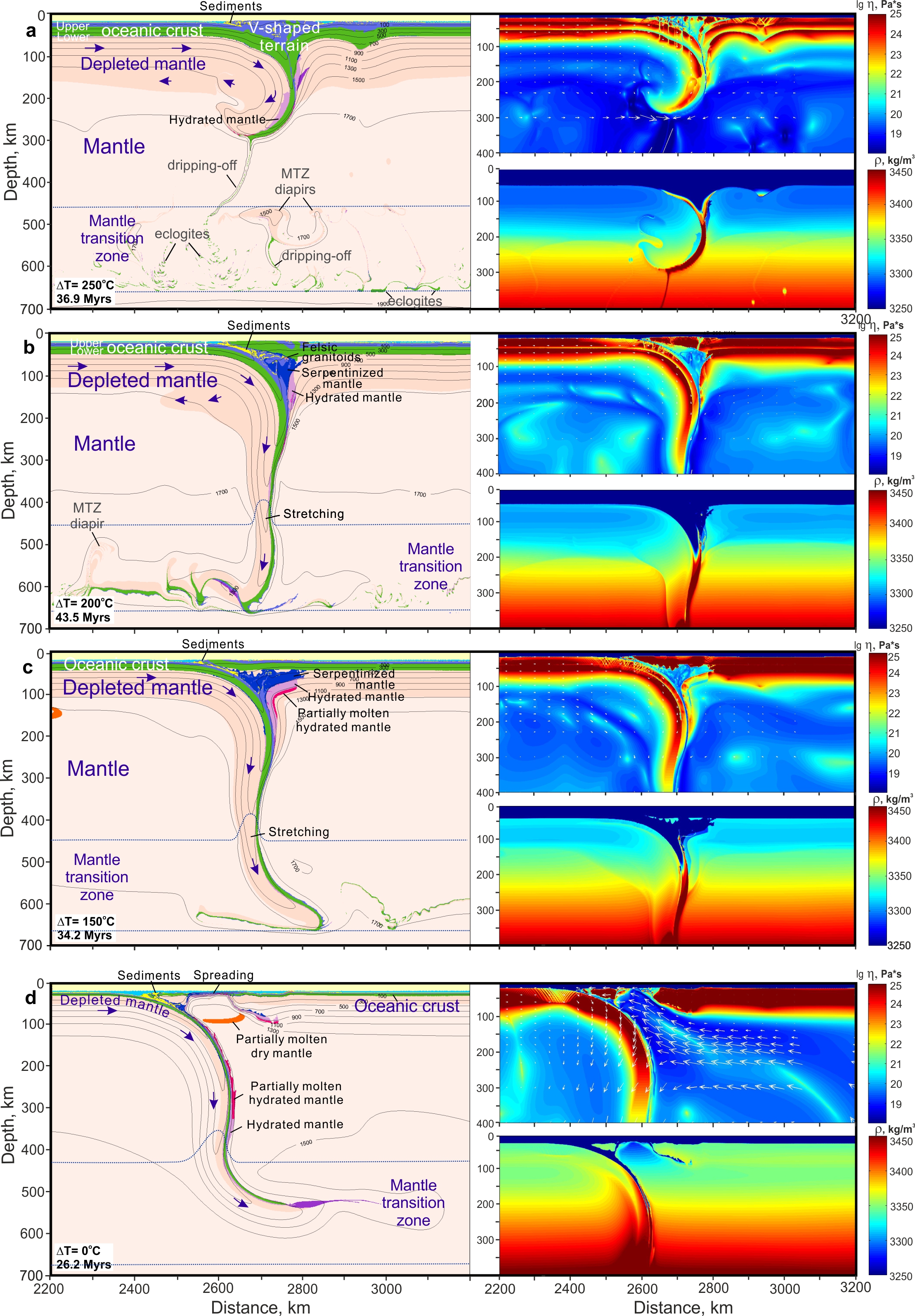


**Suppl. Fig. S3.** Changes in subduction styles at different potential mantle temperatures and velocity of 2 cm/yr. a, Two-sided subduction with formation of over-thickened depleted mantle and V-shaped terrain at elevated mantle potential temperature (T_p_ = 1550 °C; ΔT = 250 °C; 36.9 myrs from the beginning of the experiment). b, one-sided subduction with over-thickened depleted mantle produced by viscous underplating of the subducting plate and large subduction channel at elevated mantle potential temperature (T_p_) of 1500 °C (ΔT = 200 °C; 43.5 myrs from the beginning of the experiment). Note stretching of the slab at depths >~250 km. c, one-sided subduction with formation of a serpentine-bearing subduction channel in the hanging-wall mantle at elevated mantle potential temperature (T_p_ = 1450 °C; ΔT = 150 °C; 34.2 myrs from the beginning of the experiment). Note stretching of the slab at depths >~300 km. d, retreating subduction with formation of a extensional basin at modern mantle potential temperature (T_p_ = 1300 °C; ΔT = 0 °C; 26.2 myrs from the beginning of the experiment). Density and effective-viscosity fields with velocity vectors are shown for each stage as separate panels at the bottom right and at the top right, respectively. Dotted dark-blue lines in the left column indicate upper and lower boundaries of the MTZ. Arrows show direction of plate motion and viscous flow of the depleted mantle. The colour key is as in Fig. 2 and Supplementary Figure S1.


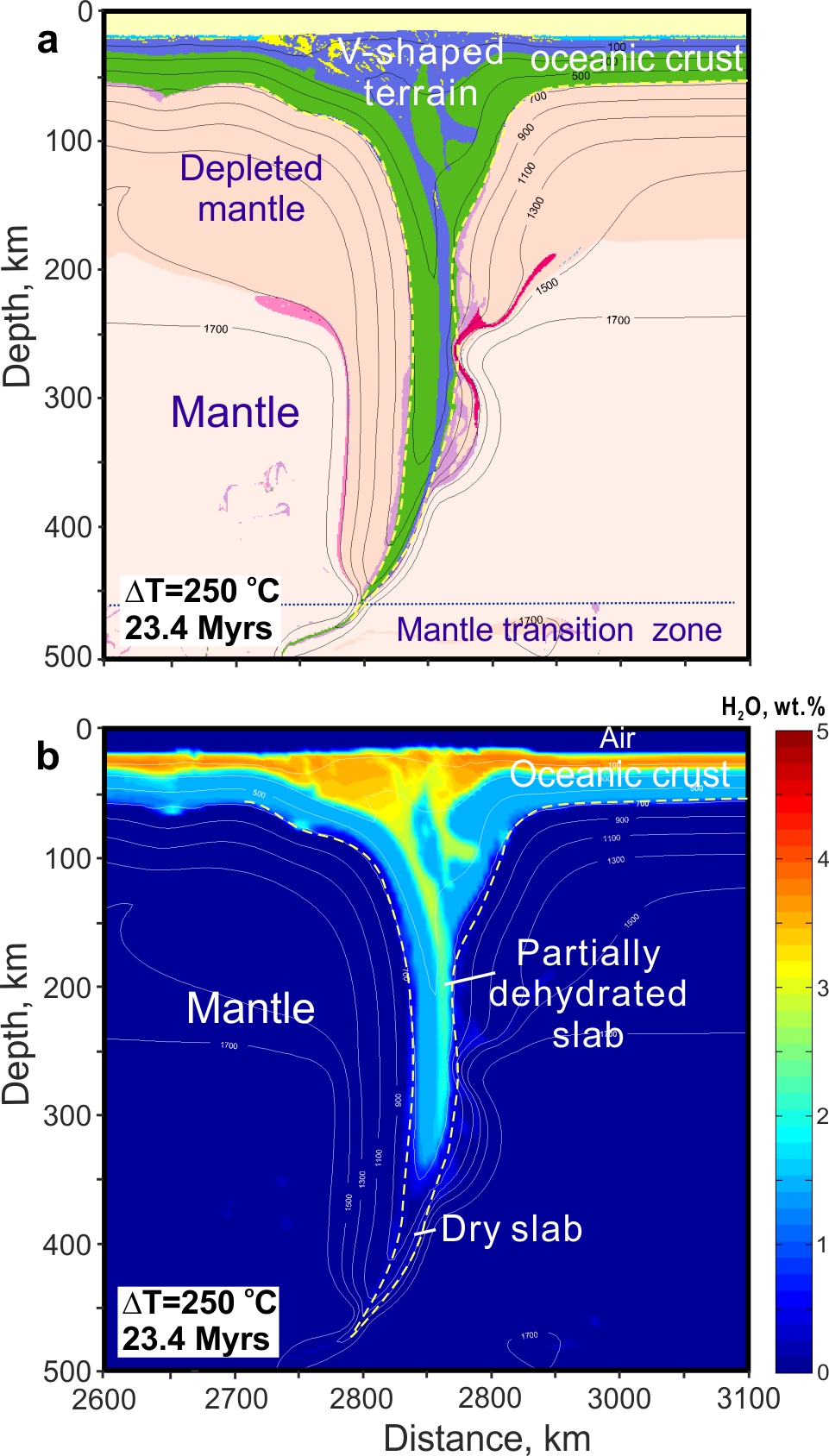


**Suppl. Fig. S4.** Drainage of the downgoing plates in a two-sided subduction zone at elevated mantle temperature (ΔT = 250 °C; T_p_ = 1,550 °C). a, Vertical and symmetrical two-sided subduction with minor hydration of the ambient mantle (23.4 Myr from the beginning of the experiment depicted at Fig. 2). b, Range of water content in the rocks (in wt %) demonstrating dehydration of the slab rocks at ≥ 150 km, becoming almost dry at ≥ 350 km. Prescribed velocity of the plate is 5 cm/yr. Dashed yellow lines indicate lower crust-mantle boundary (Moho). Dotted dark-blue line in panel a indicates the upper boundary of the MTZ. Arrows show the direction of plate motion and viscous flow of the depleted mantle. The colour key is as in Fig. 2.

**Supplementary Table S1. Conditions and results of 2D numerical experiments***

| **Model** | **Convergence rate (cm/yr)** | **Mantle potential temperature, ^o^C** |  | **Oceanic crust thickness, km (basalt/gabbro)** | **Result** | **Figure** | | |  |  |
| --- | --- | --- | --- | --- | --- | --- | --- | --- | --- | --- |
| 105 | 5 | 1,550 (Δ*T*=250 ^o^C) |  | 30(8/22) | Double-sided subduction, formation of V-shaped terrain and overthickened depleted mantle, slab break-off, small stagnant slabs, diapirs | | Fig. 2, Supplementary Data Fig. 3 | | |  |
| 106 | 5 | 1,500 (Δ*T*=200 ^o^C) |  | 25 (7/18) | One-sided subduction, formation of viscous mantle underplate and overthickened depleted mantle, slab break-off, stagnant slab. | | Fig.4 | | |  |
| 104 | 5 | 1,450 (Δ*T*=150 ^o^C) |  | 20(5/15) | One-sided subduction, large serpentinization of the mantle wedge, rare slab break-off, large stagnant slab | | Fig. 5a | | |  |
| 119 | 5 | 1,300 (Δ*T*=0 ^o^C) |  | 7 (2/5) | One-sided subduction, formation of the volcanic arc, back-arc spreading, slab motion along the mantle transition zone | | Fig. 5b | | |  |
| 115 | 10 | 1,550 (Δ*T*=250 ^o^C) |  | 30(8/22) | Double-sided subduction, formation of V-or W-shaped terrains and overthickened depleted mantle, slab break-off, small stagnant slabs, diapirs | | Supplementary Fig. S2a | | |  |
| 116 | 10 | 1,500 (Δ*T*=200 ^o^C) |  | 25 (7/18) | One-sided Z-shaped subduction, formation of overthickened depleted mantle, mantle hydration and serpentinization. | | Supplementary Fig. S2b | | |  |
| 117 | 10 | 1,450 (Δ*T*=150 ^o^C) |  | 20(5/15) | One-sided Z-shaped subduction, formation of overthickened depleted mantle, mantle hydration and serpentinization. | | Supplementary Fig. S2c | | |  |
| 118 | 10 | 1,500 (Δ*T*=200 ^o^C) |  | 7 (2/5) | One-sided Z-shaped subduction, mantle hydration and serpentinization, formation of the volcanic arc. | | Supplementary Fig. S2d | | |  |
| 120 | 5 (till 20 myrs) | 1,550 (Δ*T*=250 ^o^C) |  | 30(8/22) | During convergence: features described for the Model 105.  V-shaped terrain after switching off an imposed convergence at 20 myrs: thermal relaxation, drip of the eclogitized upper crust, formation of sodic granitoids (TTGs). | | Fig. 3 | | |  |
| 128 | 2 | 1,550 (Δ*T*=250 ^o^C) |  | 30(8/22) | Double-sided subduction, formation of V-shaped terrain and overthickened depleted mantle, dripping of the eclogitized oceanic crust of the slab. | | | Supplementary Fig. 3a | | |
| 129 | 2 | 1,500 (Δ*T*=200 ^o^C) |  | 25 (7/18) | One-sided subduction, formation of viscous mantle underplate and serpentinite-bearing subudction chanel. Stretching of the slab. | | | Supplementary Fig. 3b | | |
| 130 | 2 | 1,450 (Δ*T*=150 ^o^C) |  | 20(5/15) | One-sided subduction, serpentinite-bearing subudction chanel, stretching of the slab. | | | Supplementary Fig. 3c | | |
| 130 | 2 | 1,550 (Δ*T*=250 ^o^C) |  | 30(8/22) | One-sided subduction, slab retreat, back-arc spreading. | | | Supplementary Fig. 3d | | |

* other parameters of the numerical experiments are presented in the Supplementary Table S2

**Supplementary Table S2. Physical properties of materials used in the numerical experiments**

| **Material** | **ρ_0_^1,2^**  **(kg/m^3^)** | ***K*^3^**  **×exp(0.00004∙*P*)**  **(W/(m∙K))** | ***T*_solidus_^4,5^**  **(K, at *P* MPa)** | ***T*_liquidus_^4,5^**  **(K, at *P* MPa)** | ***H*_r_^1^**  **(μW/m^3^)** | ***H*_L_ ^1,2^**  **(kJ/kg)** | **Flow law^6^** |
| --- | --- | --- | --- | --- | --- | --- | --- |
| Sediments  Felsic crust  (upper and middle continental crust) | 2,600/2,400  (solid/ molten)  2,700/2,400  (solid/ molten) | $\left[0.64+\frac{807}{T+77}\right]$ | $889+\frac{17,900}{\left(P+54\right)}+\frac{20,200}{\left(P+54\right)^{2}}$ at *P* ≤ 1,200  831 + 0.06∙*Р* at *P* > 1,200 | 1,262 + 0.009∙*P* | 2  1.5 | 300 | Wet quartzite  *V* = 8 J/mol/MPa,  *c*_0_=10 MPa, *c*_1_=1 MPa,  γ_0_=0.15, γ_1_=0.075 |
| Basalt  (upper oceanic crust) | 3,000 (solid)  2,900 (partially molten) | $\left[1.18+\frac{474}{T+77}\right]$ | $973-\frac{70,400}{\left(P+354\right)}+\frac{77800,000}{\left(P+354\right)^{2}}$  at *P* ≤ 1,600  935+0.0035∙*Р*+0.0000062∙*P*^2^ at *P* >1,600 | 1,423 + 0.105∙*P* | 0.250 | 380 | Wet quartzite  *V* = 8 J/mol/MPa,  *c*_0_=10 MPa, *c*_1_=1 MPa,  γ_0_=0.15, γ_1_=0.075 |
| Gabbro  (lower oceanic and continental crust)l | 3,000 (solid)  2,900 (partially molten) | $\left[1.18+\frac{474}{T+77}\right]$ | $973-\frac{70,400}{\left(P+354\right)}+\frac{77800,000}{\left(P+354\right)^{2}}$  at *P* ≤ 1,600  935+0.0035∙*Р*+0.0000062∙*P*^2^ at *P*>1,600 | 1,423 + 0.105∙*P* | 0.250 | 380 | Plagioclase An_75_  *V* = 8 J/mol/MPa,  *c*_0_=10 MPa, *c*_1_=10 MPa,  γ_0_=0.6, γ_1_=0.6 |
| Dry mantle  (both fertile and depleted) | 3,390,  then P-T-depletion-dependent | $\left[0.73+\frac{1293}{T+77}\right]$ | *P*-H_2_O-dependent | *P*-H_2_O-dependent | 0.022 | 380 | Dry olivine  *V* = 8 J/mol/MPa,  *c*_0_=10 MPa, *c*_1_=10 MPa,  γ_0_=0.6, γ_1_=0.6 |
| Hydrated mantle /weak zone /serpentinized mantle | 3,390/  3,390/  3,390,  then P-T-H_2_O-dependent | $\left[0.73+\frac{1293}{T+77}\right]$ | *P*-H_2_O-dependent | *P*-H_2_O-dependent | 0.022 | 380 | Wet olivine  *V* = 8 J/mol/MPa,  *V* = 3.2 (serpentinized)  *c*_0_=10 MPa, *c*_1_=1 MPa,  γ_0_=0.1, γ_1_=0.05 |
| Melt-bearing  dry/wet mantle | 2,900/2,900  then *P-T*-depletion-dependent | $\left[0.73+\frac{1293}{T+77}\right]$ | *P*-H_2_O-dependent | *P*-H_2_O-dependent | 0.022 | 380 | Dry/wet olivine  *V* = 8 J/mol/MPa,  *c*_0_=1 MPa, *c*_1_=1 MPa,  γ_0_=0, γ_1_=0 |

**Parameters for the Flow Laws^6^**: Wet Quartzite – *A*_D_ = 10^–3.5^ MPa^–n^∙s^–1^, *n* = 2.3, *E* = 154,000 J/mol; Plagioclase An_75_ – *A*_D_ = 10^–3.5^ MPa^–n^∙s^–1^, *n* = 3.2, *E* = 238,000 J/mol; Dry Olivine – *A*_D_ = 10^4.4^ MPa^–n^∙ s^–1^, *n* = 3.5, *E* = 532,000 J/mol; Wet Olivine – *A*_D_ = 10^3.3^ MPa^–n^∙s^–1^, *n* = 4.0, *E* = 470,000 J/mol.

ρ_0_ – density, *k* – thermal conductivity, *T*_solidus, liquidus_ – solidus and liquidus temperatures, *H*_r_, *H*_L_ – heat production (radiogenic, latent), *E* – activation energy, *n* – stress component, *A*_D_ – material constant, *V* – activation volume, *T* – temperature (K), *P* – pressure (MPa). *c*_0_ and *c*_1_  – the initial and final compressive strength, γ_0_ and γ_1_ – initial and final effective friction coefficient.

**For all lithologies**: isobaric heat capacity *C*_p_ = 1,000 J/(kg∙K), thermal expansion coefficient α = 2∙10^–5^ K^–1^, and isothermal compressibility β = 0.4∙10^–2^ GPa^–1^.

**References**

1. Turcotte, D.L. & Schubert, G. Geodynamics (Cambridge Univ. Press, 2002).

2. Bittner, D., Schmeling, H. Numerical modeling of melting processes and induced diapirism in the lower crust. *Geoph. J. Int.* **123**, 59–70 (1995).

3. Clauser, C., Huenges, E. Thermal Conductivity of Rocks and Minerals. in Rock Physics and Phase Relations: A Handbook of Physical Constants (eds Ahrens, T.J.) 105–126 (AGU, Washington, DC, 685 1995).

4. Katz, R.F., Spiegelman, M. & Langmuir, C.H. A new parameterization of hydrous mantle melting. *Geochem. Geophys. Geosyst*. **4**, https://doi.org/10.1029/2002GC000433 (2003).

5. Schmidt, M. & Poli, S. Experimentally based water budgets for dehydrating slabs and consequences for arc magma generation. *Earth and Planet. Sci. Let*. **163**, 361–379 (1998).

6. Ranalli, G. *Rheology of the Earth* (Chapman & Hall, London, 1995).
